# Eddy Current Sensors Optimization for Defect Detection in Parts Fabricated by Laser Powder Bed Fusion

**DOI:** 10.3390/s23094336

**Published:** 2023-04-27

**Authors:** Romain Saddoud, Natalia Sergeeva-Chollet, Michel Darmon

**Affiliations:** Université Paris-Saclay, CEA, List, F-91120 Palaiseau, France

**Keywords:** eddy current testing, additive manufacturing, laser powder bed fusion, remote sensing, non-destructive testing, structural health monitoring

## Abstract

The production of parts by additive manufacturing is an important issue for the reduction in manufacturing costs and the creation of complex geometries. Optical inspection is often implemented in the machines during the manufacturing process in order to monitor the possible generated defects. However, it is also crucial to test the quality of the manufactured parts after their fabrication and monitor their health throughout their industrial lifetime. Therefore structural health monitoring (SHM) methods need to be studied or designed. In this paper, the eddy current method is used to control fabricated parts, as this technique is adapted to detect surface and shallow defects in conductive materials. Using simulations with the CIVA non-destructive testing software package, several sensors and their parameters were tested in order to determine the most optimal ones: a separate transmitter/receiver sensor and an isotropic sensor were finally designed. The comparison of these sensors’ efficiency was made on the detection of notches and engraved letters based on simulation and experimental tests on parts fabricated by laser powder bed fusion (L-PBF) in order to determine the optimal sensor. The various tests showed that the isotropic sensor is the optimal one for the detection and characterization of defects.

## 1. Introduction

Additive manufacturing is a technology that allows the design of parts by adding material as opposed to “traditional” processes (machining, forging, rolling, etc.), which are characterized by a removal of material (subtractive processes) [1]. Additive manufacturing has the advantage of producing complex parts using only the necessary amount of material [2].

Laser powder bed fusion (L-PBF), also known as selective laser melting (SLM), is the most commonly used additive manufacturing process for the fabrication of metal parts. It is increasingly used in various fields such as aerospace, medical, defense, and mold making [3,4].

In this process, a layer of powder is spread with the wiper, so that the laser beam selectively melts the powder. The repetition of these operations will result in the production of the final part. The different parameters such as laser power, wiper speed, and layer thickness must be optimized beforehand in order to improve the final quality of the part. For example, the use of an inappropriate laser power can lead to local overheating of the melt [5], which generally induces keyhole porosity [6]. Studies on the final mechanical properties have been carried out, and the results have shown that the parts manufactured with this process have similar or better characteristics than with a conventional process [6,7]. However, the parts’ integrity needs to be controlled at three different stages:

1. During the manufacturing process, defects such as porosities and cracks may occur [8]. An online non-destructive testing is thus required during the fabrication.
2. At the end of the additive manufacturing and before supplying the customer.
3. Throughout their industrial lifetime.

Structural health monitoring (SHM) involved in stage 3 is defined as the process of acquiring and analyzing data from on-board sensors to evaluate the health of a structure. Health is the ability to function/perform and maintain the structural integrity throughout the entire lifetime of the structure. The SHM is thus a scientific procedure composed of several non-destructive testing processes including the identification of operational and environmental loads acting on the component, the recognition of the damage caused by that loading, and the observation of damage growth as the component operates. Parts created by additive manufacturing are usually of complex geometry. When they are in service, it will be necessary to inspect critical areas subjected to important mechanical stresses for instance and in which breaking cracks can grow.

In situ monitoring technologies could be implemented to control the quality of manufactured parts layer by layer during the fabrication (stage 1) to perform the evaluation, qualification, and certification of structural parts [9] once the manufacturing process is finished (stage 2) and to monitor their health during their lifetime (stage 3).

Different techniques can be used for non-destructive testing (NDT) of metallic parts (see for instance [10,11,12,13,14,15]); they can more devoted to in-situ testing such as acoustic emission, thermography, and optical tomography. Optical emissions of the melt pool or solidified material can be detected by photodiodes, thermographic cameras, or optical tomography. This solution is intended for pores agglomeration detection of more than 0.5 mm diameter. Ultrasounds (UT) represent the most widely used technique in NDT/SHM for flaw (like notch and holes [13]) detection; but employed probes are usually in contact mode [14] or require immersion in a liquid coupling [15], and UT are not very efficient to detect small near surface flaws except in contact [14]. Eddy current testing (ET) is a good candidate for monitoring L-PBF parts. This technique integrates well into the layer-by-layer manufacturing process as well as the in-service conditions, because it is suitable for detecting surface or near-surface defects. Moreover, a SHM method using eddy currents will be contactless, which could allow inspection of part areas that are difficult to access or with signs of corrosion or roughness.

If the defect is keyhole porosity, it is recommended to use an eddy current probe with a directional dependency in order to obtain a better detectability while, for non-spherical defects (absence of fusion), it is preferable to use an absolute probe with a non-directional behavior [6]. Absolute measurement allows sensitivity to both the electromagnetic parameters of the material (electrical conductivity and magnetic permeability) and the thickness of the material.

Recent studies have demonstrated the expanding use of eddy current testing for non-destructive evaluation (NDE) in various materials and applications. For example, it has been employed for detecting defects in composite materials, including carbon fiber composites [16,17], as well as in carbon steel materials [18]. Moreover, ET is increasingly being used in additive manufacturing [19,20,21].

The flexibility of ET has also been demonstrated using flexible sensors, allowing for the detection of defects on curved surfaces [22]. Furthermore, studies have explored the use of capacitance-inductance dual modality imaging systems for NDE applications [23]. These recent studies highlight the broadening range of applications for ET, making it a promising technique for NDE in various materials and contexts.

Detecting deep defects in metallic materials remains complicated because of the skin effect. When the defect is located beyond the skin thickness, the eddy current probe cannot distinguish or even detect the defect. This skin thickness is related to the excitation frequency, the electrical conductivity, and the magnetic permeability of the material [9,24]. In view of these elements, the optimization of the sensors parameters will improve the obtained results in terms of detection.

In previous recent studies the possibility of application of different types of ET sensors commercially available [25,26] or specially manufactured [27] and their comparison have been shown. The performance of ET sensors based on magnetoresistive sensors has been evaluated on artificially introduced defects in L-PBF samples [28]. Nevertheless, in these studies simulation was not employed to help designing the probes.

Early detection of flaws is an asset for the monitoring of nuclear installations notably or of manufactured parts. Simulation is usually very helpful for designing the optimal configuration of inspection (probes geometrical parameters and location for emission and reception, number of probes, used frequency, lift-off, etc.). It enables the avoidance of experimental and expensive trials for optimizing the inspection configuration.

In this paper, a study has firstly been carried out in order to select by simulation the most suitable probes and their parameters according to the type of defect to be detected. In that purpose, an eddy current testing simulation model (integrated in CIVA NDT software platform) has been employed. This model allows for the simulation of eddy current testing responses from flaws present in a specimen [29]. Comparisons between simulations and experimental data using two sensors (a separate transmitter/receiver sensor and an isotropic sensor) with a flexible pattern using the eddy current method are proposed on 316 L stainless steel L-PBF mockups. The proposed method for sensors design and comparison has the objective to efficiently use the eddy current NDT method for flaws detection in L-PBF additive manufacturing parts.

In Part 2 of this paper, ET simulation has been used to optimize the parameters of both sensors. For this purpose, a first variation study on the lift-off for the two sensors, then on the winding direction of the receiver coils for the isotropic sensor, are presented in order to determine the optimal lift-off and the direction of manufacture of the coils. The winding direction of a coil determines the direction in which the receiver coils are wound relative to the transmitter coil, causing a change in the polarity of the coil. The winding direction is either similar to the transmitting coil or opposite [30]. Experimental results obtained on the parts issued from L-PBF fabrication with both kind of sensors are analyzed and in Part 3 compared between each other and to the simulation. The performance of used sensors for surface defects detection is also discussed.

## 2. Materials and Methods

### 2.1. Eddy Current Testing Principle

The principle of the eddy current method [31] consists of creating currents in conductive materials by using a probe composed of a transmitter (coil) and a receiver (coil or magnetic sensor). The transmitter coil generates a time-varying magnetic field. This field will create induced currents (also called eddy currents) [32] in the metallic object. These currents have a distribution that depends on several parameters: the geometry of the piece, the electrical conductivity (σ) and the magnetic permeability (µ) [33]. Eddy currents create a magnetic field opposite to the excitation field. The receiver will detect this magnetic field (reaction field), which will oppose the initial excitation field according to Lenz’s law [34].

**Figure 1:**
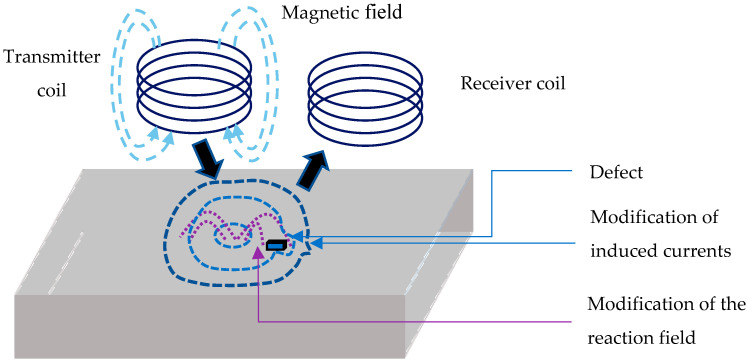
Eddy current principles with the presence of a parallelepipedic defect.

In the presence of a defect in the part to be tested (Figure 1), the current lines are deflected and the reaction field is modified [35], resulting in a variation of the voltage. Eddy current testing has been evaluated on the metal parts fabricated by L-PBF method.

### 2.2. Mock-Up Description

**Figure 2:**
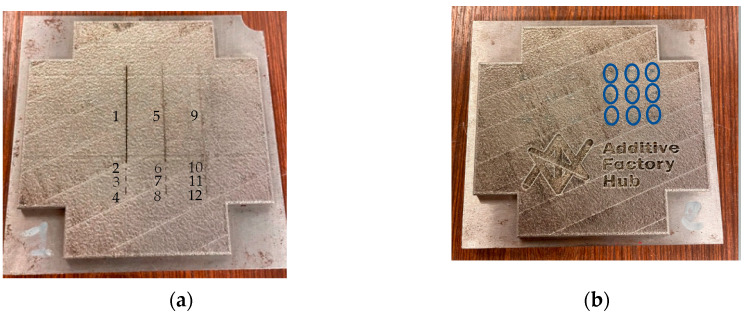
Designation of stainless steel 316 L mock-ups (**a**) with artificial defect (**b**) with nine cylindrical defects (encircled in blue) and letters printed during part manufacturing.

Two stainless steel 316 L mock-ups (Figure 2) were studied with the following dimensions: length × width × depth = 95 × 95 × 4.7 mm^3^. Measured electrical conductivity for both parts is σ = 0.8 MS/m. The employed steel is not magnetic. The first mock-up has 12 defects of different size (Figure 2a). The dimensions of the defects are indicated in Table 1. The second specimen part includes engraved letters “Additive Factory Hub” with a letter depth of 1 mm measured with a digital comparator (Figure 2b) and nine cylindrical defects obtained by electro erosion ranging from 0.15 mm diameter × 0.1 mm depth to 0.5 mm diameter × 0.5 mm depth. As an indication, the dimensions of the letter A are: 5 mm length and 5 mm width.

During fusion, lacks of fusion could be formed due to non-homogeneous powder spreading or other factors. This lack of fusion is similar to the small notches studied in this paper.

### 2.3. Sensors Optimization Using Eddy Current Simulation

CIVA is a NDT/SHM simulation and analysis platform. It includes simulation tools for different NDT techniques, notably ultrasounds [36,37], eddy currents [29], and X-rays [38,39]. Simulations with CIVA software were performed in order to optimize different parameters for the sensors, such as the air gap and the coils’ winding configuration. The model for the simulations that we have used is the model for the conductive materials, which are not issued from additive manufacturing. We assume for the simulation that the conductivity is the same for materials made by the additive manufacturing technique, and it has been proven experimentally. This simulation model is semi-analytical and is based on the volume integral equations and Green’s dyadic formalism [29].

For example, a simulation study of the air gap effect was carried out to determine the optimum distance (lift-off) between the part and the probe to obtain the maximum defect signal. For this purpose, the smallest defect in the part of Figure 2b (diameter 0.15 mm × depth 0.1 mm) was selected in simulation to optimize its detection. Moreover, for the simulations, the minimum lift-off chosen is 0.07 mm, corresponding to the thickness of one Kapton film, which needs to be placed under the sensor for its protection. This simulation was carried out in order to observe the lift-off influence on the amplitude of the defect response. Due to the roughness of the parts, it was finally necessary to place two Kapton films (0.14 mm total thickness) under the bottom of each probe in order not to damage the sensors during experiments.

#### 2.3.1. The Separate Transmitter/Receiver Sensor

**Figure 3:**
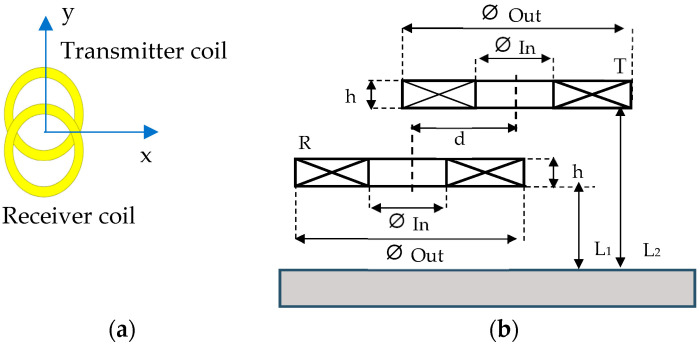
Design of the transmitter/receiver sensor. (**a**) Direction y linking the two coils centers perpendicular to the main scan direction x for experimental and simulation testing. (**b**) Front view design.

The separate transmitter/receiver sensor is an overlapping design (Figure 3) consisting of a transmitter coil and a receiver coil engraved on a Kapton flexible film. This mode means that one coil is used for excitation and another one for the reception of the signal from the part under test. This type of sensor enhances the defect signal in the impedance plane [31,40] for an homogeneous planar part. The Kapton film (thickness 0.07 mm) is used to inspect in future the parts with complex geometry, the common case of L-PBF technique fabricated parts. The lift off is not the same for the two coils because they overlap to obtain a better defect signal. Figure 3a shows the orientation (axes) of the transmitter/receiver coil for the different experimental and simulation tests.

**Figure 4:**
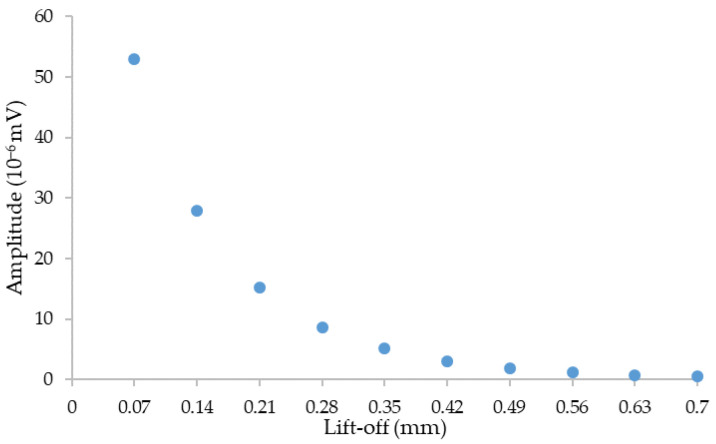
Maximum amplitude versus lift-off for a separate transmitter/receiver sensor when inspecting a cylindrical defect of dimensions Ø 0.15 mm × depth 0.1 mm at a frequency of 1 MHz.

The choice of lift-off is made from the following simulations. The curve of the maximum amplitude of the defect response signal detected by receiver coil as a function of the lift-off is given in Figure 4.

Simulation results show that an increase in lift-off leads to a decrease in signal amplitude; therefore, a sensor placed close to the mock-ups allows for a better amplitude of the defect response signal. In view of the roughness of the mock-ups, it is preferable to position the probe at 0.14 mm (two Kapton films placed under the surface of the sensor coils). Furthermore, the CIVA simulation software does not take into account the surface roughness and does not model the corresponding noise.

The geometrical parameters of the chosen coils design are given in Table 2. The lift-off coil is 0.14 mm between the part and the transmitter and 0.21 mm between the part and the receiver coil.

#### 2.3.2. The Isotropic Sensor

**Figure 5:**
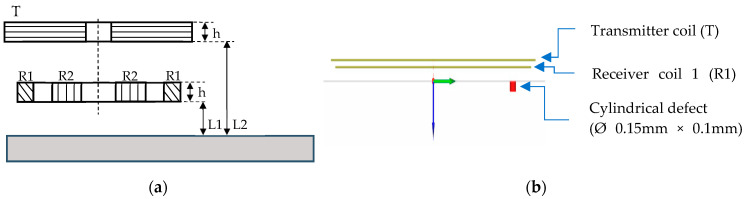
Isotropic sensor. (**a**) Scheme front view design. (**b**) Front view design in CIVA software.

The isotropic sensor pattern is composed of a transmitting coil and two receiving coils engraved on a Kapton film. A schematic representation of isotropic sensor is given in Figure 5a. This sensor has the specialty to be sensitive in two directions of the magnetic field [31].

**Figure 6:**
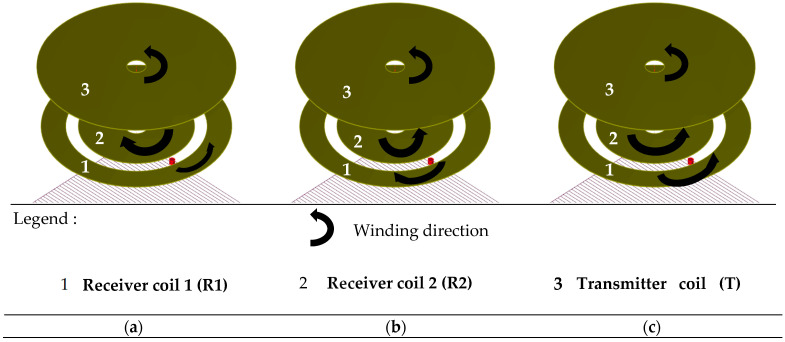
Scheme of the three winding configurations for the isotropic sensor in CIVA software. (**a**) Configuration 1. (**b**) Configuration 2. (**c**) Configuration 3.

This sensor is composed of three different winding configurations. A scheme of the three configurations is given in Figure 6.

The winding direction causes a change in the polarity of the coil. The reference winding direction is that of the transmitting coil (T); therefore, the receiving coils can be designed either in the same winding direction as the transmitting coil or in the opposite direction. Configuration 1 shows receiver coil 1 (R1) in the reference winding direction and receiver coil 2 (R2) in the opposite direction. Configuration 2 presents R1 in the reverse winding direction and R2 in the winding direction. Finally, in configuration 3, coils R1 and R2 are in the same winding direction as the transmitting coil.

**Figure 7:**
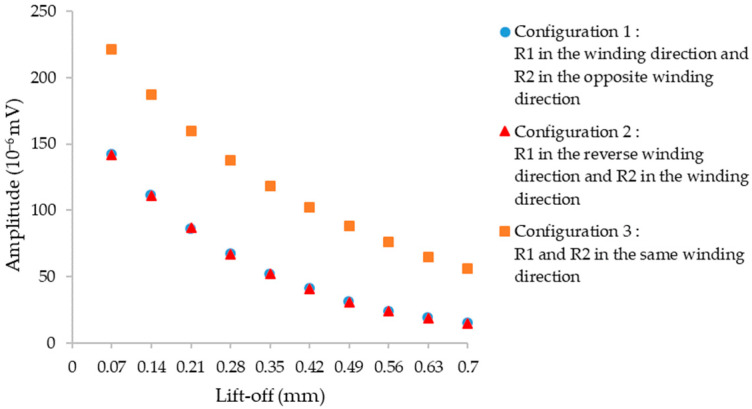
Maximal amplitude versus lift-off for the three winding configurations of the isotropic sensor with a cylindrical defect of dimension Ø 0.15 mm × 0.1 mm depth at a frequency of 1 MHz.

Different winding configurations for the coils by changing the winding direction have been tested using CIVA simulation of eddy current testing. A comparison between the three configurations has been carried out by plotting the simulated amplitude of the defect signal (of dimensions given in beginning of Section 2.3) as a function of the lift-off. The objective is to select the configuration with the maximal defect amplitude (Figure 7).

Simulation results show that configuration 3 is the configuration with the highest amplitude of the defect signal.

The optimal configuration deduced from the simulation has been chosen for the isotropic sensor design and its geometrical parameters are provided in Table 3 and Figure 5.

#### 2.3.3. Comparison of Sensors Efficiency in Simulation

**Figure 8:**
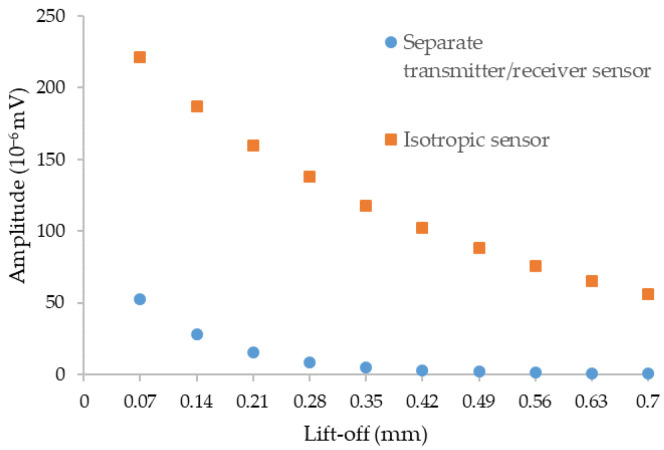
Amplitude of the maximum signal versus the lift-off for the two sensors for the inspection of a cylindrical defect of dimension Ø 0.15 mm × 0.1 mm depth at a frequency of 1 MHz.

A comparison of the influence of the defect signal amplitude versus the lift-off for the two sensors is carried out in order to determine the sensor with the highest amplitude of the defect signal but also to observe the different impact of the lift-off between the two probes. The comparison of the two curves is given in Figure 8.

According to the simulation, the isotropic sensor has the ability to obtain a higher maximum signal amplitude than the separate transmitter/receiver sensor, so it should be easier to detect defects with the isotropic sensor. In addition, the isotropic sensor is less sensitive to lift-off variation than the separate transmitter/receiver sensor (very low simulated amplitudes for important lift-off).

### 2.4. Experimental Set Up

**Figure 9:**
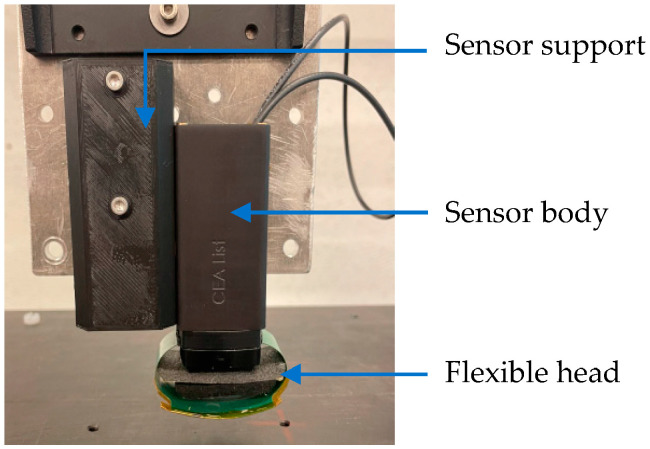
Support attached to the sensor body with the flexible head.

For experimental testing, the ET sensor (Figure 9) has been fixed on a motorized bench to carry out the displacement. The acquisition system Elotest PL300 device (Rohmann GmbH, Frankenthal, Germany) has been used during the tests. The adjustment of the phase of the emission signal has been made in order to obtain the maximum of the received signal from the part under test at the imaginary part of the signal. Thus, only the imaginary part will then be analyzed.

**Figure 10:**
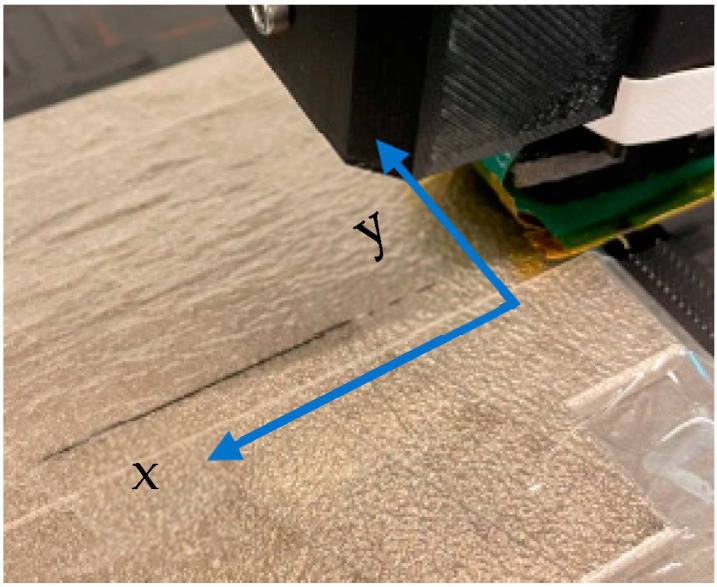
Main direction x of movement of the sensors (with incremental direction y) for the detection of notches in the AFH 01–2019 part.

The 2D image is obtained by scanning the part under test in two directions (*X* and *Y*-axis) as shown in Figure 10. Frequency choice f = 1 MHz is made in order to concentrate eddy currents on the surface of the part under test.

## 3. Results and Discussion

In order to better compare in terms of performances the two patterns, the experimental tests and simulations in CIVA software [41] have been realized. For comparison of experimental results and simulations, the signal from Ad Fa letters from Additive Factory Hub (Figure 2b) and defects of length 40 mm, 1 mm, 2 mm, and 3 mm (Figure 2a) have been simulated using the CIVA software with two patterns.

### 3.1. Defects Detection

The first part presented in Figure 2a has been tested with both sensors. The results have been then compared. The described above set-up has been used. The excitation frequency was f = 1 MHz, and the applied voltage V = 5 V, with a step size of 0.1 mm and the scan speed of 5 mm/s. The defects with a depth of less than 0.5 mm are not experimentally detected owing to the observed noise, probably due to the surface roughness; therefore, only defects for lengths of 40 mm, 1 mm, 2 mm, and 4 mm with a width of 0.3 mm and a depth of 0.5 mm will be discussed. The detected notches were simulated on the CIVA software. The direction of the scan for two sensors for the detection of the defects is shown in Figure 10.

**Figure 11:**
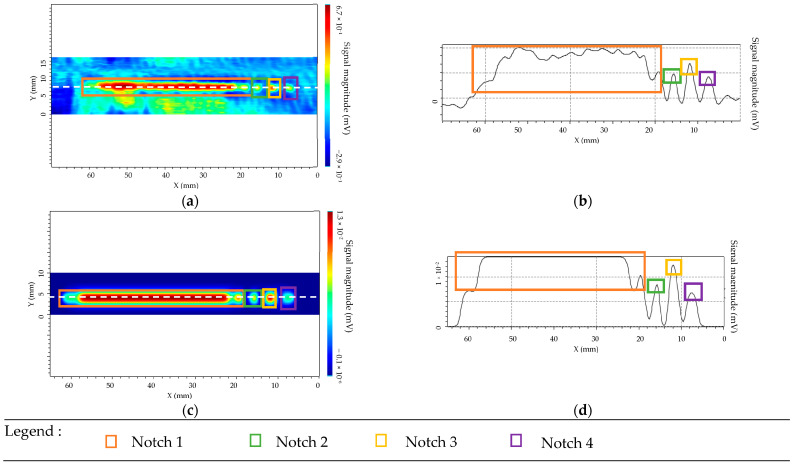
Separated T/R sensor for notch lengths of 40 mm, 1 mm, 2 mm, 4 mm with a width of 0.3 mm and a depth of 0.5 mm. (**a**) Experimental cartography with defects detection. (**b**) EC signal from the scan line represented in (**a**) with dashed line. (**c**) Simulated cartography with presence of the defects. (**d**) EC signal from horizontal cut of the scan represented in (**c**) by the dashed line.

**Figure 12:**
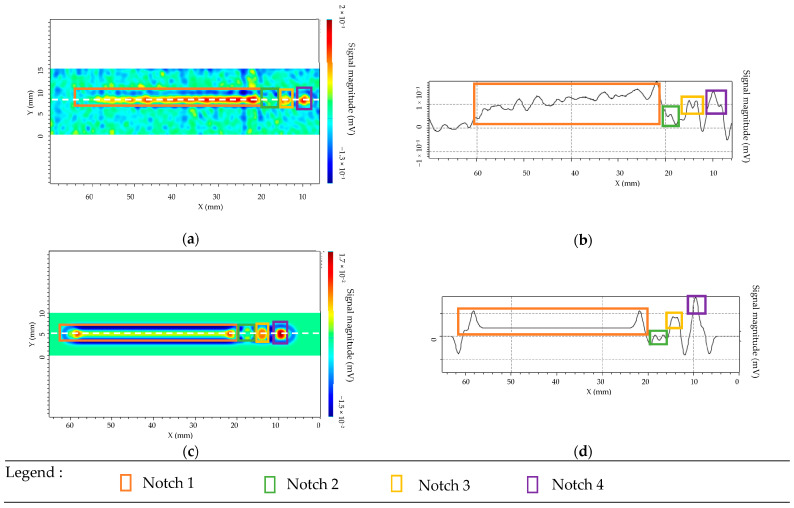
Isotropic sensor for notch lengths of 40 mm, 1 mm, 2 mm, and 4 mm with a width of 0.3 mm and a depth of 0.5 mm. (**a**) Experimental cartography with defects detection. (**b**) EC signal from the scan line represented with dashed line. (**c**) Simulation of cartography with presence of the defects r. (**d**) EC signal from horizontal cut of the scan represented by the dashed line.

Experimental and simulation results are presented in Figure 11 for the separate transmitter/receiver sensor mode and Figure 12 for isotropic sensor.

The used color codes are slightly different for the two sensors. For the transmitter/receiver sensor, the cyan blue corresponds to the background noise (near minimal signal), and the green/red shows high amplitude variation. For the isotropic sensor, green and blue coincide with the almost minimal signal and orange/red with the high amplitude variation. Four characteristic peaks corresponding to the four defects have well been observed in C-SCAN cartographies. This result has been obtained experimentally and confirmed by simulations for both patterns of sensors. For the separate sensor the simulation allows prediction with a very good agreement of the amplitude variation between each flaw. For the isotropic sensor, the simulation and experimental results are in overall good agreement, but the experimental response of the longest defect exhibits a spatially inhomogeneous behavior along the x direction: it may be due to the inhomogeneous roughness of the component, which is not taken into account in the simulation CIVA [42]. It was experimentally observed that with the separate T/R sensor, the ET signal of the defect is higher than for the isotropic sensor if the orientation of the defect corresponds to the favorable one i.e., if the defect length is along the scanning direction x (x direction in Figure 3 and Figure 8). This is confirmed for a 1 mm long defect (smallest length) with a width of 0.3 mm and a depth of 0.5 mm. The signal-to-noise ratio (SNR) for the separate transmitter/receiver sensor is 8 dB, whereas it is 5 dB for the isotropic sensor.

### 3.2. Letters Detection

The detection of letters (Figure 2b) with ET sensors has been evaluated.

**Figure 13:**
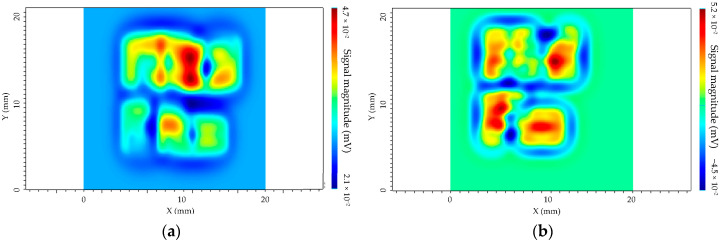
Simulation results of the spatial cartography of the Additive Factory Hub. (**a**) Separated T/R sensor, f = 1 MHz. (**b**) Isotropic sensor, f = 1 MHz.

A model of a part including the engraved letters (Ad Fa) has been created in CIVA software from several parallelepipedic defects but without disorientation to simplify the simulations. The depth of the letters is 1 mm. The spatial cartography by simulation for the two sensors is given in Figure 13.

The obtained results conclude that defects could be detected with both sensors. Moreover, the detectability for the isotropic sensor is better, due to its sensitivity in two directions of magnetic field. That means that the defects could be detected independently of their orientation. The separate transmitter/receiver sensor does not have this specificity; thus, the letters are more difficult to distinguish.

**Figure 14:**
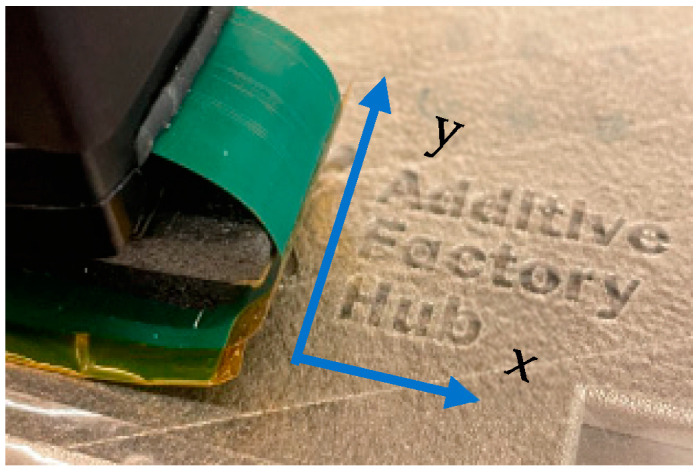
Main direction x of movement of the sensors for the detection of the letters Additive Factory Hub.

**Figure 15:**
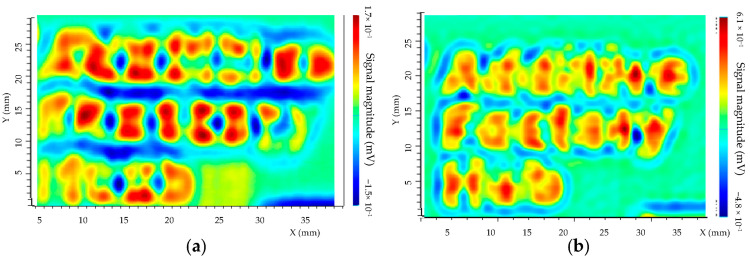
Experimental tests of the spatial cartography of the Additive Factory Hub letters of the AFH 02–2019 model. The yellow/red color represents the maximum amplitude and the green/blue color the minimum amplitude with a step size of 0.1 mm and a displacement speed of 5 mm/s. (**a**) Separated T/R sensor, f = 1 MHz. (**b**) Isotropic sensor, f = 1 MHz.

For experimental tests, the described above set-up has been used. The excitation frequency was f = 1 MHz for T/R sensor and f = 1 MHz for isotropic sensor. The applied voltage V = 5 V, the step size is 0.1 mm, and the scan speed is 5 mm/s. The directions of the scan of the two sensors for letter detection are given in Figure 14. The experimental results are shown in Figure 15.

The obtained results show that defects are detected with both sensors. For the isotropic sensor the detectability is better, due to the sensitivity in two directions of the magnetic field, as shown by simulations. The detection of letters that are not oriented along with the scan direction x of sensor is more complicated with a separate transmitter/receiver sensor. The SNR has been compared based on experimental results. It was observed that SNR for the separate transmitter/receiver sensor is 10 dB whereas for isotropic sensor the SNR is 22 dB.

### 3.3. Discussion

We have shown that large defects can be detected with the proposed technique. One perspective of this work will be to study in detail the influence of roughness on the detection of small defects (of less than 1 mm size). Here are given preliminary experimental results to approximately quantify the effect of a standard roughness for L-BPF manufacturing technique.

**Figure 16:**
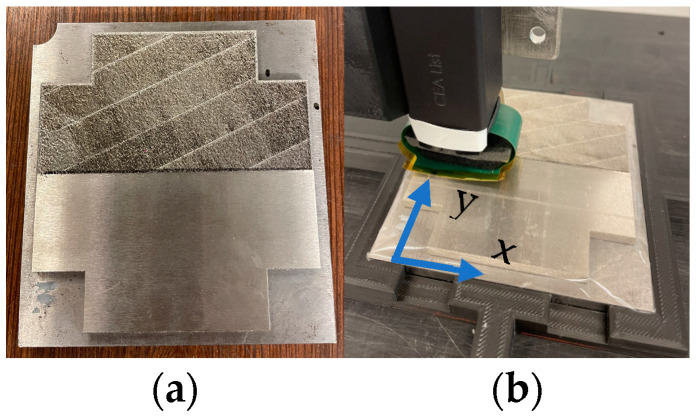
Designation of stainless steel 316 L mock-up for roughness analysis (**a**) with a smooth polished surface and a rough surface; (**b**) main direction x of movement of the sensors (with incremental direction y).

The mock-up composed of both a smooth polished surface and a rough surface and the directions of scan of the two sensors is given in Figure 16.

**Figure 17:**
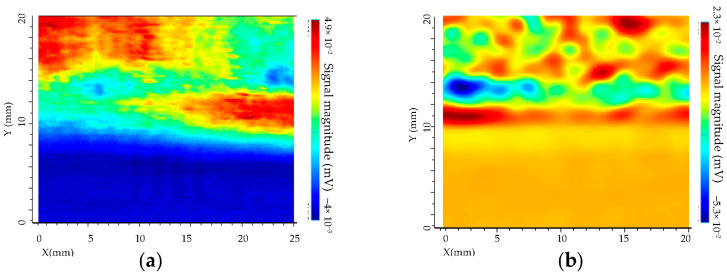
Experimental tests of the spatial cartography of the roughness. The yellow/red color represents the maximum amplitude and the green/blue color the minimum amplitude with a step size of 0.1 mm and a displacement speed of 5 mm/s. (**a**) Separated T/R sensor, f = 1 MHz. (**b**) Isotropic sensor, f = 1 MHz.

Experimental results are presented in Figure 17 for the separate transmitter/receiver sensor and isotropic sensor.

Brief analysis of the experimental results shows that the received signal is well homogenous on the smooth polished surface and that heterogeneous and important amplitude variations are observed on the rough surface. This observation lets us suppose a complexity of detection for small defects of size of the same order compared to roughness.

## 4. Conclusions and Future Work

A comparison between two eddy current sensors, a separate transmitter/receiver sensor and an isotropic sensor, has been carried out on 316 L stainless steel parts. The sensors and corresponding inspection configuration have been designed thanks to simulations. The mock-ups were manufactured using additive manufacturing with the L-PBF process by incorporating letters during the process and artificial defects. In addition, a comparison between the experimental and simulation tests has been carried out to observe a correlation between these tests. The comparison results show that the spatial resolution and also the SNR are better with the isotropic sensor for letters detection. For defect detection, the transmitter receiver sensor is better in terms of SNR, since the defects have been placed along the scanning direction (preferential orientation for eddy current detection). It is, however, finally preferable to use the isotropic sensor for detection of defects with unknown orientation.

For future work, further optimization of the sensors could be investigated to achieve detection of smaller flaws. In addition, analyses of the parts roughness can be performed in order to differentiate a defect from the roughness. Finally, experimental tests will be carried out on real defects. If the detection results are conclusive, the proposed sensors could then be integrated into an L-PBF machine. In addition, for integration during in-service conditions, the dynamic system tested here using a mechanical scanning should be replaced by a static one with an electronic scanning; for that, multielement probes using the previously studied probes as repeating units have to be investigated.

## Figures and Tables

**Table 1:** Dimensions of the 12 notches of the AFH 01–2019 model part of Figure 2a.

Description	1	2	3	4	5	6	7	8	9	10	11	12
Length (mm)	40	1	2	3	40	1	2	3	40	1	2	3
Width (mm)	0.3											
Depth (mm)	0.5				0.2				0.1			

**Table 2:** Geometrical parameters of transmitter/receiver sensor.

Parameters	Transmitter Coil (T)	Receiver Coil (R)
Inner diameter (⌀ In) (mm)	4	
Outer diameter (⌀ Out) (mm)	5.12	
Height (h) (µm)	10	
Number of spires	4	
Lift-off (mm)	(L_2_) 0.21	(L_1_) 0.14
Offset distance (d) between the two coil centers (mm)	2	

**Table 3:** Geometrical parameters of the isotropic sensor.

Parameters	Receiver Coil 1 (R1)	Receiver Coil 2 (R2)	Transmitter Coil (T)
Inner diameter (mm)	3.64	0.6	0.5
Outer diameter (mm)	4.92	3	5.15
Height (µm)	10		
Number of spires	4	8	15
Lift-off (mm)	0.14		0.21

## Data Availability

The data were obtained at CEA List.
